# Functionalization-induced changes in the structural and physical properties of amorphous polyaniline: a first-principles and molecular dynamics study

**DOI:** 10.1038/srep20621

**Published:** 2016-02-09

**Authors:** X. P. Chen, Q. H. Liang, J. K. Jiang, Cell K. Y. Wong, Stanley Y. Y. Leung, H. Y. Ye, D. G. Yang, T. L. Ren

**Affiliations:** 1 Key Laboratory of Optoelectronic Technology & Systems, Education Ministry of China, Chongqing University and College of Opto-electronic Engineering, Chongqing University, Chongqing 400044, China; 2 Faculty of Electromechanical Engineering, Guilin University of Electronic Technology, 541004 Guilin, China; 3 Institute of Microelectronics, Tsinghua University, 100084 Beijing, China; 4 Changzhou Institute of Technology Research for Solid State Lighting, 213161 Changzhou, China

## Abstract

In this paper, we present a first-principles and molecular dynamics study to delineate the functionalization-induced changes in the local structure and the physical properties of amorphous polyaniline. The results of radial distribution function (RDF) demonstrate that introducing -SO_3_^−^Na^+^ groups at phenyl rings leads to the structural changes in both the intrachain and interchain ordering of polyaniline at shorter distances (≤5 Å). An unique RDF feature in 1.8–2.1 Å regions is usually observed in both the interchain and intrachain RDF profiles of the -SO_3_^−^Na^+^ substituted polymer (i.e. Na-SPANI). Comparative studies of the atom-atom pairs, bond structures, torsion angles and three-dimensional structures show that EB-PANI has much better intrachain ordering than that of Na-SPANI. In addition, investigation of the band gap, density of states (DOS), and absorption spectra indicates that the derivatization at ring do not substantially alter the inherent electronic properties but greatly change the optical properties of polyaniline. Furthermore, the computed diffusion coefficient of water in Na-SPANI is smaller than that of EB-PANI. On the other hand, the Na-SPANI shows a larger density than that of EB-PANI. The computed RDF profiles, band gaps, absorption spectra, and diffusion coefficients are in quantitative agreement with the experimental data.

Polyaniline holds a special position amongst the family of conducting polymers because of its unique and novel properties^1^ with wide potential applications in micro/nano devices^2^,^3^,^4^, supercapacitors^5^, nanomaterials^6^, solar cells^7^, color displays^8^, and polymer membranes^9^. Introducing ionizable groups at rings or nitrogen sites might lead to new properties of parent polyaniline without substantially sacrificing conductivity and new opportunities in polyaniline-based materials and devices^10^,^11^,^12^. Direct functionalization to polyaniline backbone with ionizable groups such as boronic acid^13^, phosphoric acid^14^, sulfonic acid^10^,^15^, and acrylic acid^16^ have been reported. In general, two effects (stereo and electronic) in the local structure of polyaniline are associated with the substituent of ionizable groups^10^. The change in the stereo structure results in the decrease of the conjugation in the *π* system, the hypsochromic shifts of the band-to-band transition feature of π-π* electrons in the UV-vis spectra, and the decreased stability of polysemiquinone radical cation^10^,^15^. The change in electronic structures primarily leads to the increase of solubility in basic aqueous solutions, the pH dependence of the conductivity, charge localization, and the self-doping mechanism^10^,^11^,^12^,^13^,^15^. For instance, Epstein *et al*.^15^ have demonstrated that the sulfonated structure of polyaniline has improved the pH-dependent conductivity over the range of 0 ≤ pH ≤ 14 with a lower activation energy for the conductivity.

Although previously studied works^9^,^10^,^11^,^16^,^17^,^18^,^19^,^20^,^21^ have greatly contributed to the understanding of the molecular and physical properties of the conventional polymer system within the polyaniline family, such as emeraldine base of polyaniline (EB-PANI) and its conducting form, a number of fundamental issues remain to be uncovered. Maron and Winokur^18^ have investigated the processing-induced changes in the molecular structure of EB-PANI by RDF analysis of X-ray scattering data, but the effect of functionalization on the molecular structure of amorphous polyaniline in terms of intrachain and interchain ordering is not yet investigated. Venkataraman *et al*.^22^ have found that the conductance of *π*-conjugated biphenyl systems is linearly related to the cos^2^*Ф* of the inter-ring. A similar trend in increased conductance values has been reported for the series biphenyldithiols (BPDTs) with a divided *π*-system^23^. Yet, how does the functionalization, such as ring-substituted, affect the torsion angles between the adjacent rings in the case of polyaniline remains to be uncovered. The chain conformation and film morphology^24^, the location of functional groups and secondary guest species^25^, and the chain-to-chain packing^26^ often influence the manifested properties of polymers. However, knowledge on the role that functional groups play in affecting the various physical properties (e.g. eletcronic and optical) of polymers at atomic level is still unclear^19^. Furthermore, polyaniline is a potential material for gas separation, and experimental study^18^ indicated that in the presence of water, the EB matrix has covalently bonded to either carbon or nitrogen atoms. Introducing ionized groups such as -SO_3_^−^, -SO_3_^−^Na^+^,-COO^−^ and -COO^−^Na^+^ on polyaniline chains^27^,^28^, hydrogen-bond may form in the polymer matrix. Therefore, the effect of the hydrogen-bond network on gas transport properties of polymers remains to be theoretically validated.

In the present paper, the above issues are addressed through the analysis of the effect of ionizable group -SO_3_^−^Na^+^ on the local structure and physical properties (electronics, optical and gas transport properties) of amorphous polyaniline by employing a combination of first-principles and molecular dynamics techniques. The polymers considered in this work are EB-PANI and its ionizable group -SO_3_^−^Na^+^ ring-substituted form which is named as Na-SPANI. To understand how the ionizable groups -SO_3_^−^Na^+^ at phenyl rings affect the local structure of polyaniline, both the intrachain and interchain RDF profiles of the two polymers are calculated and analysed. In order to further delineate the intrachain change, the atom-atom pairs (types and distances), bond structures (bond lengths and angles) and the torsion angles impact on the structural changes of the polyaniline are also investigated. In addition, results of band gap and absorption spectra are presented to provide insight into both the electronic and optical properties. Furthermore, the self-diffusivity of water vapor in the two polymers is estimated to describe the change of gas transport property of EB-PANI at the presence of the -SO_3_^−^Na^+^ groups. To our knowledge, this work is the first attempt to compute the effect of ionizable group on the structural and physical properties of polyaniline at atomic and molecular levels.

## Result and Discussions

**Figure 1:**
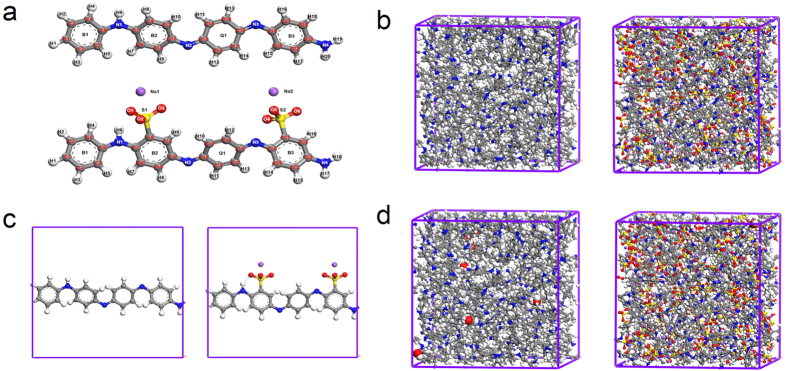
(**a**) The non-periodic models of EB-PANI (top) and Na-SPANI (bottom) with one monomer used for the calculation of optical properties; (**b**) The periodic molecular models for RDF calculations of EB-PANI (left) and Na-SPANI (right); (**c**) The periodic models used for structural comparison and electronic properties calculations of EB-PANI (left) and Na-SPANI (right) where the blue dotted line represents the hydrogen-bond between oxygen and hydrogen atoms in the intrachain packing; (**d**) The periodic models for the water diffusion study of EB-PANI (left) and Na-SPANI (right), where hydrogen, carbon, nitrogen, oxygen, sulfur and sodium atoms are shown in white, gray, blue, red, yellow and violet colors, respectively.

The non-periodic models of EB-PANI and Na-SPANI with one monomer are given in Fig. 1a and used for the prediction of optical properties of the polymers. The periodic models for the investigation of the structural (RDF profiles, atom-atom pairs, bond structures, and torsion angles) and physical (electronic and gas transport) properties of Na-SPANI as well as comparative studies with the parent EB-PANI are shown in Fig. 1b–d, respectively.

### Radial distribution function

In statistical mechanics, RDF in a system of polymers gives a measure of the probability that there will be an atom located in a spherical shell of infinitesimal thickness at a distance, *r*, from the reference atom. The resulting function is often expressed in terms of *G*(*r*) and, described by^18^


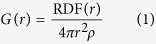


where *ρ* = *N*/*V* is the overall number density of atoms; *N* is the total number of atoms and *V* is the volume of the polymer system. Therefore, the RDF is normalized by *G*(*r*). According to the definition by Hansen and McDonald^29^, RDF *G*(*r*) can be calculated as follows:


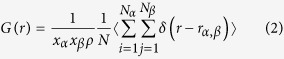


where the atoms of chemical types *α* and *β* are separated by 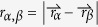, 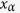
and 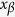 respectively denote the mole fraction of chemical type *α* and *β*, 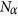 and 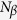 respectively represent the number of atoms of chemical type *α* and *β*, *δ*(*r*) is the Dirac delta function, and 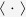 is the ensemble average. The prime indicates that terms where *i* = *j* are excluded when the chemical types are the same.

As given by Eqs (1) and (2), the RDF is related to the pair distribution function *G*(*r*). *G*(*r*) can be decomposed into the sum of an intrachain *G*_I_(*r*) and of an interchain *G*_E_(*r*)^18^. *G*_I_(*r*) provides information on intrachain correlations between atoms covalently bonded in a given polymeric chain while *G*_E_(*r*) gives details about interchain correlations on the chain packing.

**Figure 2:**
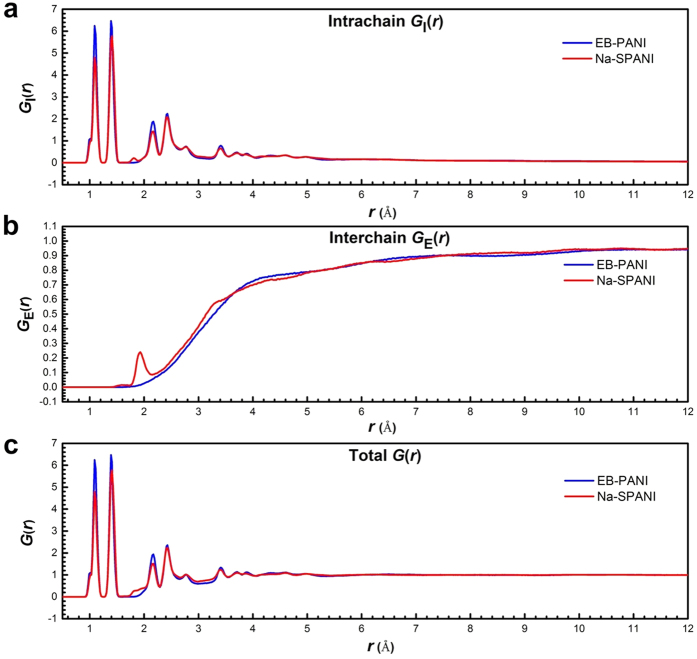
Results for RDF calculation of EB-PANI and Na-SPANI explicitly comparative showing (**a**) the intrachain, (**b**) the interchain, and (**c**) the total (interchain + intrachain) G(*r*).

The calculated *G*_I_(*r*), *G*_E_(*r*) and *G*(*r*) profiles for each of polymer systems are shown in Fig. 2a–c, respectively. In Fig. 2a, the positions of the peaks of the *G*_I_(*r*) profiles for EB-PANI and Na-SPANI are mostly coinciding. It is of interest to note that the relative *G*_I_(*r*) intensity fluctuations of Na-SPANI samples at *r* ≤ 4.0 Å are less than those of EB-PANI. It is also of interest to note that a small peak at *~*1.8 Å is only presented in the *G*_I_(*r*) of Na-SPANI. This weak peak represents the intrachain hydrogen-bond as illustrated in Fig. 1c using blue dotted line. From Fig. 2b, a unique peak at 1.93 Å is observed in the *G*_E_(*r*) profiles of Na-SPANI while no distinct peak is presented in that of EB-PANI. This noteworthy difference is attributed to the hydrogen-bond between the -SO_3_^−^Na^+^ group and the H atoms on neighboring polymer chains. The presence of the -SO_3_^−^Na^+^ groups also induces oscillations in the interchain *G*_E_(*r*) spectra in the 2.1–3.4 Å region which gives higher *G*_E_(*r*) intensity in the Na-SPANI^30^. The result implies that with the introduction of -SO_3_^−^Na^+^ groups on the polyaniline chain, the hydrogen-bond and van der Waals are induced and therefore the interactions among polymer chains at *r* ≤ 3.4 Å are improved. Using the intrachain *G*_I_(*r*) data in Fig. 2a together with the interchain *G*_E_(*r*) data in Fig. 2b for each of the polymer, it is possible to derive the total *G*(*r*) profiles as given in Fig. 2c. It is most important to note that, whether in the intrachain *G*_I_(*r*) profiles or in the total *G*(*r*) profiles at *r* ≤ 4.0 Å, the intensity fluctuations of the major peaks of Na-SPANI are less than those of EB-PANI. It is also important to note that at 1.8 ≤ *r* ≤ 2.0 Å the relative total *G*(*r*) intensity fluctuations of Na-SPANI is significantly improved by the presence of the -SO_3_^−^Na^+^ group. This can be attributed to the presence of -SO_3_^−^Na^+^ groups on the phenyl rings results in the generation of new hydrogen-bond in both the intrachains and interchains. To make a quantitative comparison between the computed and measured RDF, in Table 1 we compare the positions of all the major peaks in the computed distributions with the experimental data given by Maron and Winokur^18^. The good agreement between the computed and measured RDF data confirms the reality and accuracy of our atomistic models.

### Structure comparison

After reaching equilibrium conditions, the molecular configuration of a single polymer chain is fixed. Using *ab initio* calculations, the interatomic distances between atoms (as given in Fig. 1a) have been listed in Table S1 of the supplementary information. From the supplementary information, it is clearly seen that the functional groups have hardly any effect on the bond length of C-H. Generally speaking, except the 1.1 Å, 1.8 Å and 2.1 Å peaks, no significant interatomic distances differences have been observed between the EB-PANI and Na-SPANI molecules. The first peak centered at 1.09 Å is observed in both the polymers represents the primary C-H intrachain pairs^18^, such as C_2_–H_2_ (see Fig. 1a). Lower intensity of this peak at 1.09 Å for Na-SPANI is observed. This may indicate weaker intrachain interaction of the C-H is possessed in the Na-SPANI structure. A peak at 1.81 Å only exists in the Na-SPANI model. The peak represents the C-S and O-H interaction of the SO_3_^−^Na^+^ groups with the neighbor atoms. A better illustration of the O-H interaction of the Na-PANI molecules is shown with the blue dotted line in Fig. 1c. The peak at 2.17 Å represents the secondary C-H (e.g. C_3_-H_5_) and the multilevel H-H (e.g. H_4_-H_6_ or H_5_-H_7_) intrachain pairs in PANI. With the substitution of the H atoms with the -SO_3_^−^Na^+^ groups, C-H and H-H groups are less in Na-SPANI. This explains the reduction of intensity of this peak for Na-SPANI as shown in Fig. 2a. With the increase of the distances, there are modest changes in atom-atom pair distances between EB-PANI and Na-SPANI due to the presence of the functional groups. Some representative atom-atom pair distances have been picked out, as listed in Table 2. Bond angle (*δ*) is an important parameter to reflect the intrachain structure of polymer chain. Therefore, the bond angles of the backbone for the two polymers are calculated, and the representative bond angles are summarized in Table 3. It is evident from Table 3 that the difference in the bond angles the two structures (EB-PANI and Na-SPANI) is infinitesimally small.

**Figure 3:**
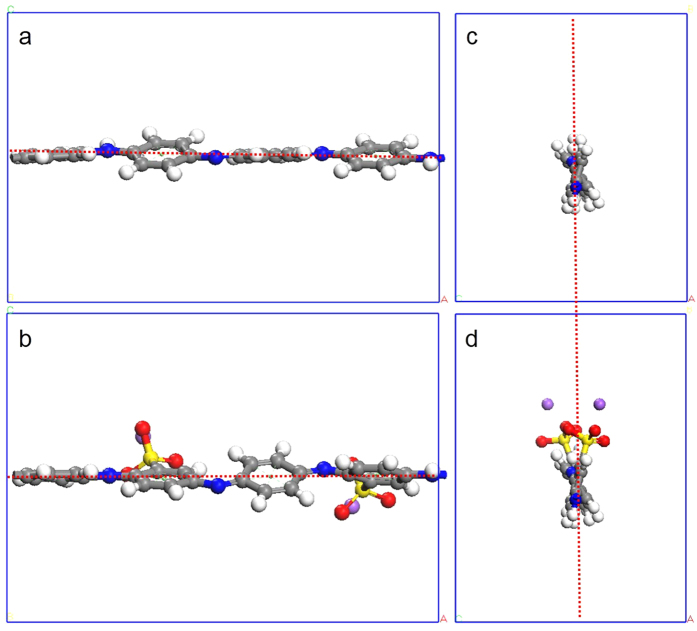
Comparison of 3D structures of EB-PANI and Na-SPANI using periodic molecular models (**a**,**b**) top view; (**c**,**d**) side view. The red dotted line is the central plane as reference.

In order to further understand how the derivatization at rings alters the spatial structure of polyaniline, the torsion angles between the adjacent two rings for the two polymers have also been calculated, as listed in Table 3. The functional groups induced variations (∆*Φ*) of these torsion angles are also calculated and listed in Table 3. It is noticeable that there is surprising difference in the torsion angles, especially in the torsion angles between phenyl and quinone, for EB-PANI and Na-SPANI. It is also noticeable that the absolute values of torsion angles are close to each other in the case of EB-PANI while the absolute values of *Φ*_C17-C18-C19-C21_ exhibit significant difference as compared with other torsion angles in Na-SPANI. In other words, EB-PANI has much better interchain ordering than that of Na-SPANI. To have a good overview on the local structure change, the three-dimensional (3D) structures in equilibrium of the polymers are illustrated in Fig. 3. From the top views of the structure as given in Fig. 3a,b, it has been found that the angles between the ring plane and the reference plane (red dotted line) of Na-SPANI has been significantly changed as compared with that of EB-PANI. This phenomenon can be also seen from the side view of the optimized 3D model structure as shown in Fig 3c,d. From this, it is confirmed that the adjacent phenyl rings of the polymer have larger torsion angles with respect to the plane of the nitrogen atoms due to the possible steric repulsion between the SO_3_^−^Na^+^ groups and hydrogens on the adjacent phenyl rings.

### Electronic properties

Polyaniline is an intrinsically conductive polymer. According to the Mott’s theory, at a given temperature, the density of states (DOS) at the Fermi energy *N*(E) determined the dc conductivity of polyaniline can be calculated as follows^31^:


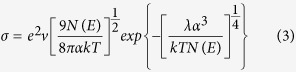


where *σ* is the dc conductivity; *e*, *ν*, *N*(E) represent the electronic charge, the hopping frequency and the DOS at the Fermi level, respectively; and *α*, *k* and *T* denote the inverse rate of the fall of the wave function, Boltzmann’s constant and the thermodynamic temperature, respectively; *λ*(≈18.1) is a dimensionless constant.

**Figure 4:**
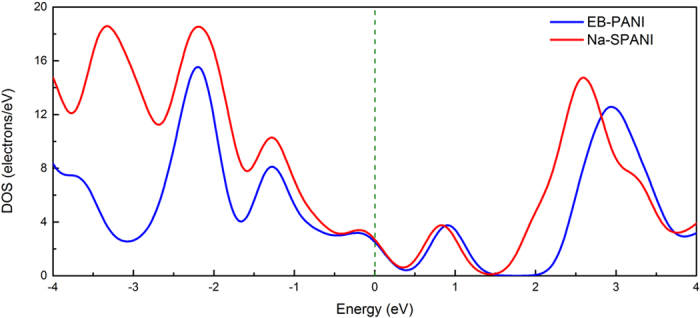
Density of states of EB-PANI and Na-SPANI monomer under the periodic condition.

Towards a better understanding of the effect of the -SO_3_^−^Na^+^ group on the conductivity of polyaniline, we firstly calculate the band gap of the two polymers. The band gaps of EB-PANI and Na-SPANI are 1.067 eV and 1.099 eV, respectively. It is implicit in the results of band gaps that introducing -SO_3_^−^Na^+^ group on ring slightly changes the conductivity of EB-PANI. In addition, we investigate how the DOS depend on the functionalization at ring. And the DOS of EB-PANI and Na-SPANI are compared in Fig. 4. It is remarkable to find that the dominant features of the DOS at the Fermi level, *N*(E) exhibited by the vertical dashed line, remain very similar. Meanwhile, from Fig. 4 we notice that the DOS of Na-SPANI at the left side of the Fermi level *N*(E) is higher than that of EB-PANI, which means more valence bands have been introduced in Na-SPANI. We also notice that the DOS of Na-SPANI at right side of the Fermi level *N*(E) has a slightly left shift as compared with that of EB-PANI. The shift may be due to a contribution from the Na^+^ ion. Both observations are the direct consequence of the presence of the -SO_3_^−^Na^+^ group on the phenyl rings. As mentioned before, -SO_3_^−^Na^+^ groups on the polyaniline backbone change the torsional angles between adjacent rings, therefore, the charge transport in Na-SPANI systems would be discounted. But the results of *N*(E) and DOS indicate that derivatization at ring does not substantially change the electronic properties of the parent polyaniline.

### Absorption spectra

**Figure 5:**
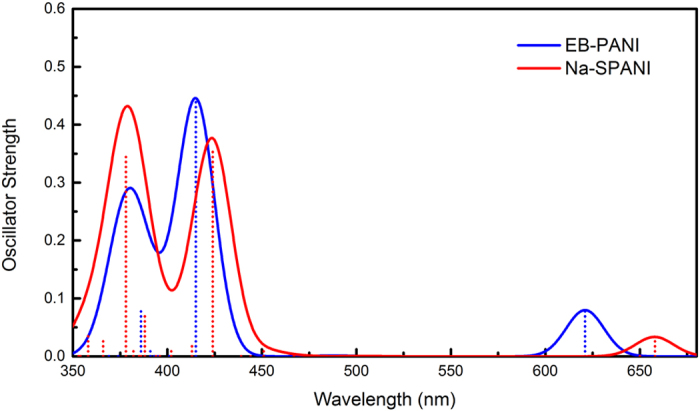
UV−vis spectra of EB-PANI and Na-SPANI.

Figure 5 shows the UV–vis spectra of EB-PANI and Na-SPANI. The electronic spectra of EB-PANI consist of three major absorption bands, which are centered at 380 nm, 415 nm, and 621 nm, respectively. The first absorption band at 380 nm is ascribed to *π*-π* electronic transition, and the third absorption band at 621 nm are ascribed to the “exciton” transition based on earlier experimental studies on the polyaniline system^10^,^32^. When the absorption occurred in intrachain, the excitation leads to formation of a “molecular” exciton (positive charge on adjacent benzenoid units bound to the negative charge centered on the quinoid), while interchain charge transfer from the highest occupied molecular orbital (HOMO centered on the benzenoid rings) to the lowest unoccupied molecular orbital (LUMO centered on the quinoid rings) may result in the formation of positive and negative^10^. Two similar adsorption bands respectively centered at 326 nm and 620 nm are observed from the experiment work did by McCall *et al*.^32^. Comparing the predicted and measured data, two differences are obsereved: i) the first absorption band in the predicted absorption spectra has shifted somewhat due to the limitation the oligomer length; ii) The oscillator strength of our modeling are smaller than that of the experiment^32^, this may be attributed to the local changes reflected by the exciton transition, which are sensitive to a chain conformation related process since strong solvatochromism. The peak values of the EB-PANI dissolved in NMP (N-methyl-2-pyrrolidinon) reported by Yue *et al*.^10^ are also greatly smaller than the peak values reported by McCall *et al*.^32^ After introducing -SO_3_^−^Na^+^ groups on the polyaniline backbone, comparing with the adsorption peaks of EB-PANI, the first absorption band of Na-SPANI located at 378 nm shows a hypsochromic shift. This absorption band is also assigned to the *π*-π* electronic transition and the hypsochromic shift caused by the presence of -SO_3_^−^Na^+^ groups that increases the torsional angles and decreases the conjugation of the polymer system. This phenomenon is also agree with the band gap of Na-SPANI is bigger than that of EB-PANI. The other absorption band appeared at 657 nm basically coincide with the experimental value ~620 nm is also assigned to the exciton transition. This is due to the -SO_3_^−^Na^+^ groups are strong electron withdrawing groups, the groups would decrease the electron density of the benzene, therefore less electron excitation and the required energy will be reduced, the peak shows bathochromic shift. Whether from the position and strength of the major absorption bands, the introduction of -SO_3_^−^Na^+^ groups on the phenyl rings has obviously changed the optical properties of the parent EB-PANI. It is of interest to note that a distinct at ~420 nm has been observed in both absorption spectra of EB-PANI and Na-SPANI. What caused this phenomenon is still unknown.

### Diffusion coefficient

The governing physics of small molecule transport in polymer matrix is described along with one-dimensional (1D) analytical solutions of related transport processes. The mean square displacement (MSD) function, which increases linearly with time in a liquid or a gaseous phase system with free motion of atoms, is used to calculate the diffusion coefficient *D*:


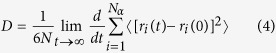


where 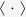 represents an average over all the guest molecules, *r*_*i*_(*t*) and *r*_*i*_(0) denote the position vector of the analyte molecule *i* in space at time *t* and its initial position, respectively.

**Figure 6:**
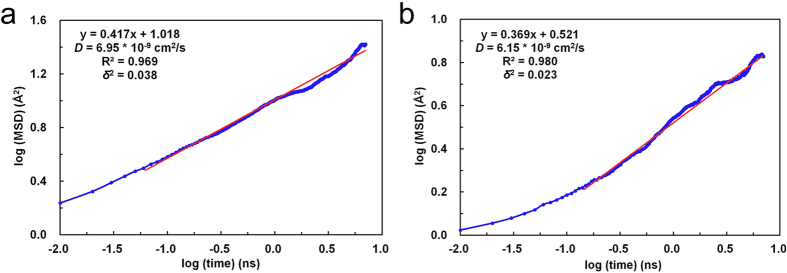
Plot of log (MSD) against log (time) of H_2_O diffusion in (**a**) EB-PANI and (**b**) Na-SPANI.

The gas transport properties of polyaniline for gas separation application are highly dependent on the molecular structures. The diffusion properties of water in the polymers are quantified. Using the molecular models given in Fig. 1d, the MSD of water molecules in EB-PANI and Na-SPANI systems have been calculated. Figure 6a,b plot log (MSD) versus log (time) of the diffusion of water in EB-PANI and Na-SPANI respectively. The plots show a linear relations between log (MSD) and log (t) which mean the diffusive transport are up to equilibrium (with the correlation coefficient *R*^2^_EB-PANI_ = 0.969 and *R*^2^_Na-SPANI_ = 0.980 then the variances *δ*^2^_EB-PANI_ = 0.038 and *δ*^2^_Na-SPANI_ = 0.023, respectively in the two models). The diffusion coefficients of water in the EB-PANI and Na-SPANI are calculated by Eq. (4), with the *D*_EB-PANI_ = 6.95 × 10^−9^ cm^2^/s and *D*_Na-SPANI_ = 6.15 × 10^−9^ cm^2^/s. To verify the models, we compare the calculated values with the experimental data for the diffusion of water vapor in HCl and HBr doped polyaniline as reported by Ostwal *et al*.^33^ The measured diffusion coefficients are *D*_PANI–Cl_ = 3.14 × 10^−9^ cm^2^/s and *D*_PANI–Br_ = 2.43 × 10^−9^ cm^2^/s, respectively. The computed and measured diffusion coefficients are in the same order of magnitude. The good agreement between simulation results and experimental data has confirmed the accuracy of our models again. The computed diffusion coefficients of water in EB-PANI are larger than that of Na-SPANI. We observe that after complete equilibrium, the density of model polymer EB-PANI is 1.23 g/cm^3^ which agree well with experiment value (1.245 ± 0.006 g/cm^3^)^21^,^34^, and the density of model polymer Na-SPANI is 1.44 g/cm^3^. The density of Na-SPANI is larger than that of EB-PANI. This implies the -SO_3_^−^Na^+^ substituent will reduce the open morphology in model polymer, and finally results in the decrease of the self-diffusivity of water, which is consistent with the result of the diffusion coefficients.

## Conclusion

In conclusion, a combination of first-principles and molecular dynamics techniques has been used to more fully investigate the functionalization-induced changes in the local structure and physical properties within polyaniline polymers. The results for EB-PANI and its -SO_3_^−^Na^+^ substituted form Na-SPANI are particularly inspiring because both the intrachain and interchain RDF’s can be independently modeled and distinguished. And the computed RDF profiles show a good agreement with the experimental data given by Maron and Winokur^18^. We observe that an unique RDF feature in 1.8–2.1 Å regions is usually resolved in both the interchain and intrachain RDF profiles of the resulting substituted polyaniline (i.e. Na-SPANI) and this indicates the -SO_3_^−^Na^+^ groups have covalently bonded to either hydrogen or nitrogen atoms in the polymer matrix. Further evidences of irreversible changes in the intrachain ordering are observed from the structure comparison analysis including atom-atom pairs, bond structures, torsion angles and 3D structures. It shows that EB-PANI has much better intrachain ordering than that of Na-SPANI. Besides, band gap, DOS and absorption spectra of each polymer are calculated to provide insight into both the electronic and optical properties and to link the structure change from the macroscopic level. The result of band gap and DOS show that derivatization at ring slightly change the electronic properties of the parent polyaniline. In contrast, obvious changes in the optical properties of Na-SPANI as compared with EB-PANI are observed from the absorption spectra. Furthermore, the effect of the -SO_3_^−^Na^+^ substituent on the gas transport properties of polyaniline has also been investigated. The computed diffusion coefficient of water in Na-SPANI exhibits more than ten percentage (~13%) of decrease than the one of EB-PANI. And the Na-SPANI shows a larger density than that of EB-PANI. It is inferred that the presence of -SO_3_^−^Na^+^ groups has reduced the open morphology in model polymer, and finally results in the decrease of the self-diffusivity of water. Comparative studies of the structural and physical properties show similarities due to the same backbone structure and differences because of the -SO_3_^−^Na^+^ groups on the phenyl rings. Our findings provide insight into the role that the -SO_3_^−^Na^+^ group plays in affecting the structural, electronic, optical and gas transport properties in the polyaniline system. It is clear that the functional groups play a critical role in controlling the material properties of polyaniline. Therefore, we are of interest to study the effects of different derivatization at ring and nitrogen sites with different functional groups on the transport, solubility, electronic, chemical and optical properties of polyanilines in the coming further study. Furthermore, we are also planning to work on potential micro/nano devices based on some new polyaniline with tailored material properties.

## Molecular Models and Computational Details

### Polymers

The monomer structures for EB-PANI and Na-SPANI were given in Fig. 1a. All the polymer chains were generated by using the polymer builder based on its stereoisomerism (tacticity) and sequence isomerism (connectivity). The tacticity of the repeat units in the constructed polymer is isotactic. The side groups could be on one side (isotactic), alternate sides (syndiotactic) or randomly arranged (atactic) around the polymer backbone. The torsion angle in degrees between new repeat units is generated randomly in the range: 180 to –180. The connectivity of the monomer units was head-to-tail^27^.

### RDF calculations

To estimate the RDF *G*(*r*) of the polymers, eight polymer chains were assembled into a 3D unit cell with a low initial density of 0.80 g/cm^3^ by using the “Amorphous Cell” module^21^ in the software of Materials Studio^®^ 7.0 (Accelrys, San Diego, CA, USA), and each polymer chain had 10 monomers. To suppress surface effects while keeping the number of particles in the model of reasonable size, periodic boundary condition (PBC), in which the system was considered to be surrounded on all sides by replicas of itself, was employed^27^. 3696 atoms in the cubic box of 34 Å for the EB-PANI system and 4336 atoms in cubic box of 37 Å for the Na-SPANI system were used. The periodic atomistic models for the two polymers were given in Fig. 1b. The polymer models were initially minimized by MM. This was followed by compressing at high pressures 1 GPa using isothermal-isobaric ensemble (NPT) for 20 ps and then decompressed at pressure 0.5 GPa for 50 ps and 0.0001 GPa for 200 ps respectively to make the density (*ρ*) of polymers as close as possible to their experimental value at 298 K. This was then followed by running annealing dynamics using a stepwise procedure of canonical ensemble (NVT), in which the system temperature was varied in cycles from one temperature to another and back again. In our case, temperatures were varied in increments of 50 K for a cycle from 298–698 K and back, and each step was carried out for 50 ps. In order to overcome energy barriers and to create a final structure with low-potential-energy characteristics, five cycles of the annealing dynamics were performed. This was then followed by a NPT dynamics at 1 atm and 298 K, and in duration of 1000 ps. Ultimately, the computed density of EB-PANI system is 1.22 g/cm^3^ which was reasonably close to the experimental density of 1.30 g/cm^3^ 35, this suggesting that the density of 1.49 g/cm^3^ for Na-SPANI was reliable. Subsequently, a NVT dynamics at 298 K was run for 7 ns, and the output of the dynamics was used to calculate the RDF of polymer system.

### Structure comparison calculations

The periodic atomistic models given in Fig. 1c were used to study the effect of -SO_3_^−^Na^+^ groups on the molecular structure including pair correlations (type and distance), bond structures (length and angle), torsion angles and 3D structures. All the models were generated using one polymer chain with one monomer surrounded by vacuum. The structure comparison calculations were performed by using DMol^3^ code of Materials Studio 7.0, which was an *ab initio* quantum mechanical program employing density functional theory (DFT)^36^. The exchange-correlation approximations was treated by the standard Perdew-Burke-Ernzerhof (PBE) generalized gradient approximation (GGA) with long range dispersion correction which used the Grimme’s scheme^37^. A double numerical basis set plus polarization basis sets (DNP) were used. The *k*-point was set to 12 × 1 × 1. A tighter smearing criterion of 0.005 hartree was applied to ensure accurate electronic convergence. Self-consistent field procedure was carried out with a convergence criterion of 10^−6^ a.u.

### Electronic properties calculations

In order to investigate the electronic properties of these two polyaniline at a more real condition, we run a canonical ensemble-molecular dynamics (NVT-MD) at room temperature (298 K) using the Forcite package of Materials Studio after the structure comparison calculations^38^. The total simulation time was 100 ps and the time step was 1.0 fs, the commercial force field “COMPASS” was used to evaluate the atomic forces. The temperature was controlled by the “Berendsen” method using a half-life for decay to the target temperature in 0.1 ps. The non-bonded electrostatic and van der Waals forces were controlled by “Ewald” at “Fine” quality and the summation methods “atom-based” with a cutoff value of 10.5 Å, respectively.

### Absorption spectra calculations

To investigate the optical properties of EB-PANI and Na-SPANI, a monomer model of these two polymers were geometry optimized by DMol^3^ code under the non-periodic environment, respectively. The set parameters were the same with the structure comparison calculations except for the k-point was set to Gamma.

### Diffusion coefficient calculations

To estimate the self-diffusivity of water in EB-PANI and Na-SPANI, ten water molecules were added to the simulation box containing 80 monomers, as shown in Fig. 1d. The energy of the atomistic models was then minimized by MM. The polymer models were then compressed and equilibrated/relaxed using the same methodology as addressed for RDF. Subsequently, NVT-MD simulations at 298 K were carried out for 7 ns, and the diffusion coefficients were then calculated from the MSD of the water molecules. All the MM and MD simulations for RDF and diffusion coefficient calculations were performed on Forcite Plus in Materials Studio 7.0. The second generation forcefield of Condensed-phase Optimized Molecular Potentials for Atomistic Simulation Studies (COMPASS) was employed. The COMPASS forcefield provided more accurate precision in determining the polymer properties and had been verified by our previous work^21^. All MD simulations were run with a 1.0 fs time step. The temperature and pressure were controlled by the Berendsen’s method using a half-life for decay to the target temperature of 0.1 ps (decay constant) and 0.1 ps for the pressure scaling constant. “Ewald” and “Atom based” summation methods were respectively used to calculate the electrostatic and van der Waals terms, the cut-off distance was 15.5 Å and the algorithm was smart.

## Additional Information

**How to cite this article**: Chen, X. P. *et al*. Functionalization-induced changes in the structural and physical properties of amorphous polyaniline: a first-principles and molecular dynamics study. *Sci. Rep*. **6**, 20621; doi: 10.1038/srep20621 (2016).

## Supplementary Material

Supplementary Information

## Figures and Tables

**Table 1:** The positions of the major peaks (in Å) of the computed total *G*(*r*) in Fig. 2(c) and their comparison with the experimental data from Maron and Winokur^18^.

EB-PANI		Na-SPANI
Computed	Measured^18^	Computed
1.09		1.09
1.39	1.40 ± 0.01	1.41
	1.8 (Hydrogen-atom terms)	1.81(Hydrogen-atom terms)
2.17		2.17
2.43	2.44 ± 0.02	2.43
2.77	2.90 ± 0.05	2.77
3.41	3.33 ± 0.10	3.41
3.71	3.74 ± 0.05	3.71
3.89		3.87
4.33	4.22 ± 0.05	4.33
4.59	4.56 ± 0.10	4.59
4.97	4.93 ± 0.10	4.97

**Table 2:** Comparisons of the representative atom-atom pair distances (in Å) between EB-PANI and Na-SPANI.

Pair distance			
EB-PANI		Na-SPANI	
*d*_C7-C9_	1.424	*d*_C7-C9_	1.439
*d*_C23-N4_	1.405	*d*_C23-N4_	1.409
*d*_H5-H7_	2.134	*d*_H5-H7_	2.078
*d*_H4-H6_	2.235	*d*_H4-H6_	2.170
*d*_H10-H11_	2.154	*d*_H9-H10_	2.027
*d*_H12-H14_	2.448	*d*_H13-H14_	2.548
*d*_C14-C18_	2.508	*d*_C14-C18_	2.447
*d*_C14-C15_	2.443	d_C14-C15_	2.501
*d*_C21-N3_	2.377	d_C21-N3_	2.421
*d*_C13-H13_	3.458	d_C13-H12_	3.262
*d*_C3-H4_	3.838	d_C3-H4_	3.735
*d*_H12-H15_	4.393	*d*_H11-H14_	4.668
*d*_C17-C21_	4.592	*d*_C17-C21_	4.471

**Table 3:** Comparisons of bond angles and torsion angles in EB-PANI and Na-SPANI.

EB-PANI		Na-SPANI		
Structure	*δ* (°)	Structure	*δ* (°)	Δ*δ* (°)
∠C_4_-C_6_-N_1_	117.613	∠C_4_-C_6_-N_1_	116.457	−1.156
∠C_5_-C_6_-N_1_	126.547	∠C_5_-C_6_-N_1_	127.813	1.266
∠**C**_**6**_**-N**_**1**_**-C**_**7**_	136.934	∠C_6_-N_1_-C_7_	137.417	0.483
∠**C**_**7**_**-C**_**9**_**-C**_**12**_	123.053	∠C_7_-C_9_-C_12_	123.676	0.623
∠**C**_**11**_**-N**_**2**_**-C**_**13**_	132.275	∠C_11_-N_2_-C_13_	132.386	0.111
∠**C**_**18**_**-N**_**3**_**-C**_**19**_	132.78	∠C_18_-N_3_-C_19_	133.062	0.282
∠**C**_**23**_**-N**_**4**_**-C**_**1**_	137.529	∠C_23_-N_4_-C_1_	137.951	0.422
**Structure**	***Φ***_***EB-PANI***_ **(°)**	**Structure**	***Φ***_***Na-SPANI***_ **(°)**	**Δ*Φ*** **(°)**
**C**_**5**_**-C**_**6**_**-C**_**7**_**-C**_**9**_	−151.903	C_5_-C_6_-C_7_-C_9_	−158.205	6.302^a^
**C**_**10**_**-C**_**11**_**-C**_**13**_**-C**_**14**_	149.812	C_10_-C_11_-C_13_-C_14_	−160.252	49.936^b^
**C**_**17**_**-C**_**18**_**-C**_**19**_**-C**_**21**_	−156.016	C_17_-C_18_-C_19_-C_21_	127.209	76.775^b^
**C**_**22**_**-C**_**23**_**-C**_**1**_**-C**_**2**_	158.316	C_22_-C_23_-C_1_-C_2_	−165.636	36.048^b^

a: Δ*Φ*(°) = **|***Φ*_*EB−PANI*_**|**−**|***Φ*_*Na-SPANI*_|.

b: Δ*Φ*(º) = (180−|*Φ*_*EB–PANI*_|) + (180−|*Φ*_Na-SPANI_|).
