# Supplementary material for: Functionalization-induced changes in the structural and physical properties of amorphous polyaniline: a first-principles and molecular dynamics study
Source: Sci Rep. 2016 Feb 9;6:20621. doi: 10.1038/srep20621 (PMC4746652; doi:10.1038/srep20621)
Supplement: Supplementary Information [file srep20621-s1.pdf]

Supplementary Information

Functionalization-induced changes in the structural and physical properties of amorphous polyaniline: a first-principles and molecular dynamics study

X. P. Chen, [1,2,*] Q. H Liang,[2] J. K. Jiang,[2] C. K.Y. Wong,[4] Stanley Y.Y. Leung,[4] H. Y. Ye,[3,4] D. G. Yang[2] and T. L. Ren[1,*]

[1]*Institute of Microelectronics, Tsinghua University, 100084 Beijing, China*

[2]*The Faculty of Electromechanical Engineering, Guilin University of Electronic Technology, 541004 Guilin, China*

[3]*Electronic Components, Technology and Materials, Delft University of Technology, Delft 2628 CD, The Netherlands*

[4] *Changzhou Institute of Technology Research for Solid State Lighting, Changzhou 213161, China*

*Correspondence should be addressed to X.P.C. (Tel.:+86 10 62782712; Fax: +86 10 62771130; E-mail: xianpingchen@tsinghua.edu.cn) or T.L.R. (Tel.: +86 10 6278 2712; Fax: +86 010 6277 1130; E-mail: RenTL@tsinghua.edu.cn)

Supplementary Table S1 Comparisons of the atom-atom pair types and distances (in Å) between EB-PANI and Na-SPANI.

Pair correlations					
Peak	EB-PANI		Peak	Na-SPANI	
	$d_{C2\text{-}H2}$	1.088		$d_{C2\text{-}H2}$	1.088
	$d_{C3\text{-}H3}$	1.093		$d_{C3\text{-}H3}$	1.093
	$d_{C4\text{-}H4}$	1.093		$d_{C4\text{-}H4}$	1.092
	$d_{C5\text{-}H5}$	1.089		$d_{C5\text{-}H5}$	1.088
	$d_{C8\text{-}H7}$	1.089		$d_{C8\text{-}H7}$	1.089
	$d_{C9\text{-}H8}$	1.094		$d_{C10\text{-}H8}$	1.092
	$d_{C10\text{-}H9}$	1.091		$d_{C12\text{-}H9}$	1.087
1.09	$d_{C12\text{-}H10}$	1.09	1.09	$d_{C14\text{-}H10}$	1.089
	$d_{C14\text{-}H11}$	1.091		$d_{C15\text{-}H11}$	1.092
	$d_{C15\text{-}H12}$	1.092		$d_{C16\text{-}H12}$	1.091
	$d_{C16\text{-}H13}$	1.092		$d_{C17\text{-}H13}$	1.091
	$d_{C17\text{-}H14}$	1.09		$d_{C20\text{-}H14}$	1.092
	$d_{C20\text{-}H15}$	1.09		$d_{C22\text{-}H15}$	1.094
	$d_{C21\text{-}H16}$	1.091		$d_{C22\text{-}H15}$	1.087
	$d_{C22\text{-}H17}$	1.094			
	$d_{C24\text{-}H18}$	1.089			
	$d_{C6\text{-}N1}$	1.413		$d_{C6\text{-}N1}$	1.411
	$d_{C7\text{-}N1}$	1.406		$d_{C7\text{-}N1}$	1.39
	$d_{C11\text{-}N2}$	1.383		$d_{C11\text{-}N2}$	1.382
	$d_{C13\text{-}N2}$	1.334		$d_{C13\text{-}N2}$	1.337
	$d_{C18\text{-}N3}$	1.335		$d_{C18\text{-}N3}$	1.325
	$d_{C19\text{-}N3}$	1.382		$d_{C19\text{-}N3}$	1.372
	$d_{C23\text{-}N4}$	1.405		$d_{C23\text{-}N4}$	1.409
	$d_{C1\text{-}C2}$	1.41		$d_{C1\text{-}C2}$	1.412
	$d_{C2\text{-}C4}$	1.399		$d_{C2\text{-}C4}$	1.398
	$d_{C1\text{-}C3}$	1.419		$d_{C1\text{-}C3}$	1.42
	$d_{C3\text{-}C5}$	1.399		$d_{C3\text{-}C5}$	1.399
	$d_{C4\text{-}C6}$	1.418		$d_{C4\text{-}C6}$	1.42
	$d_{C5\text{-}C6}$	1.411		$d_{C5\text{-}C6}$	1.412
	$d_{C7\text{-}C8}$	1.415		$d_{C7\text{-}C8}$	1.419
	$d_{C7\text{-}C9}$	1.424		$d_{C7\text{-}C9}$	1.439
1.39	$d_{C8\text{-}C10}$	1.395	1.41	$d_{C8\text{-}C10}$	1.394
	$d_{C9\text{-}C12}$	1.393		$d_{C9\text{-}C12}$	1.395
	$d_{C10\text{-}C11}$	1.43		$d_{C10\text{-}C11}$	1.431
	$d_{C11\text{-}C12}$	1.428		$d_{C11\text{-}C12}$	1.425
	$d_{C13\text{-}C14}$	1.454		$d_{C13\text{-}C14}$	1.455
	$d_{C13\text{-}C15}$	1.46		$d_{C13\text{-}C15}$	1.46
	$d_{C15\text{-}C17}$	1.373		$d_{C15\text{-}C17}$	1.373
	$d_{C16\text{-}C18}$	1.46		$d_{C16\text{-}C18}$	1.46
	$d_{C16\text{-}C14}$	1.373		$d_{C16\text{-}C14}$	1.373
	$d_{C17\text{-}C18}$	1.454		$d_{C17\text{-}C18}$	1.453

	$d_{\mathrm{C19-C20}}$	1.428		$d_{\mathrm{C19-C20}}$	1.426
	$d_{\mathrm{C19-C21}}$	1.43		$d_{\mathrm{C19-C21}}$	1.434
	$d_{\mathrm{C20-C22}}$	1.392		$d_{\mathrm{C20-C22}}$	1.393
	$d_{\mathrm{C21-C24}}$	1.395		$d_{\mathrm{C21-C24}}$	1.397
	$d_{\mathrm{C22-C23}}$	1.425		$d_{\mathrm{C22-C23}}$	1.422
	$d_{\mathrm{C23-C24}}$	1.414		$d_{\mathrm{C23-C24}}$	1.411
				$d_{\mathrm{O1-H6}}$	1.884
			1.81	$d_{\mathrm{S1-C9}}$	1.787
				$d_{\mathrm{S2-C21}}$	1.792
	$d_{\mathrm{H5-H7}}$	2.134		$d_{\mathrm{H5-H7}}$	2.078
	$d_{\mathrm{H4-H6}}$	2.235		$d_{\mathrm{H4-H6}}$	2.17
	$d_{\mathrm{H6-H8}}$	2.211		$d_{\mathrm{H9-H10}}$	2.027
	$d_{\mathrm{H10-H11}}$	2.154		$d_{\mathrm{H15-H17}}$	2.188
	$d_{\mathrm{H14-H15}}$	2.1		$d_{\mathrm{C11-H9}}$	2.195
	$d_{\mathrm{H17-H20}}$	2.189		$d_{\mathrm{C11-H8}}$	2.155
	$d_{\mathrm{C11-H9}}$	2.151		$d_{\mathrm{C6-H5}}$	2.181
	$d_{\mathrm{C11-H10}}$	2.185		$d_{\mathrm{C1-H3}}$	2.169
	$d_{\mathrm{C6-H5}}$	2.177		$d_{\mathrm{C1-H2}}$	2.183
	$d_{\mathrm{C1-H3}}$	2.168		$d_{\mathrm{C4-H2}}$	2.138
	$d_{\mathrm{C1-H2}}$	2.178		$d_{\mathrm{C5-H3}}$	2.14
	$d_{\mathrm{C4-H2}}$	2.143		$d_{\mathrm{C6-H4}}$	2.164
	$d_{\mathrm{C5-H3}}$	2.14		$d_{\mathrm{C3-H5}}$	2.141
	$d_{\mathrm{C6-H5}}$	2.177		$d_{\mathrm{C2-H4}}$	2.14
	$d_{\mathrm{C3-H5}}$	2.146		$d_{\mathrm{C7-H7}}$	2.172
	$d_{\mathrm{C2-H4}}$	2.141		$d_{\mathrm{C8-H8}}$	2.148
	$d_{\mathrm{C6-H4}}$	2.167		$d_{\mathrm{C9-H9}}$	2.121
2.17	$d_{\mathrm{C7-H8}}$	2.169	2.17	$d_{\mathrm{C10-H7}}$	2.14
	$d_{\mathrm{C7-H7}}$	2.178		$d_{\mathrm{C13-H11}}$	2.167
	$d_{\mathrm{C8-H9}}$	2.151		$d_{\mathrm{C13-H10}}$	2.208
	$d_{\mathrm{C9-H10}}$	2.14		$d_{\mathrm{C15-H13}}$	2.138
	$d_{\mathrm{C10-H7}}$	2.147		$d_{\mathrm{C14-H12}}$	2.143
	$d_{\mathrm{C12-H8}}$	2.143		$d_{\mathrm{C16-H10}}$	2.121
	$d_{\mathrm{C9-H10}}$	2.14		$d_{\mathrm{C18-H13}}$	2.198
	$d_{\mathrm{C13-H12}}$	2.166		$d_{\mathrm{C17-H11}}$	2.142
	$d_{\mathrm{C13-H11}}$	2.202		$d_{\mathrm{C18-H12}}$	2.168
	$d_{\mathrm{C14-H13}}$	2.14		$d_{\mathrm{C19-H14}}$	2.17
	$d_{\mathrm{C15-H14}}$	2.126		$d_{\mathrm{C20-H15}}$	2.142
	$d_{\mathrm{C16-H11}}$	2.129		$d_{\mathrm{C21-H16}}$	2.125
	$d_{\mathrm{C17-H12}}$	2.14		$d_{\mathrm{C23-H16}}$	2.192
	$d_{\mathrm{C18-H13}}$	2.165		$d_{\mathrm{C22-H14}}$	2.147
	$d_{\mathrm{C18-H14}}$	2.204			
	$d_{\mathrm{C19-H15}}$	2.189			
	$d_{\mathrm{C19-H16}}$	2.15			
	$d_{\mathrm{C20-H17}}$	2.142			
	$d_{\mathrm{C22-H15}}$	2.136			
	$d_{\mathrm{C24-H16}}$	2.151			
	$d_{\mathrm{C23-H18}}$	2.18			
	$d_{\mathrm{C23-H17}}$	2.17			

	$d_{\text{C21-H18}}$	2.144				
	$d_{\text{H2-H4}}$	2.428			$d_{\text{H2-H4}}$	2.425
	$d_{\text{H3-H5}}$	2.431			$d_{\text{H3-H5}}$	2.424
	$d_{\text{H8-H10}}$	2.437			$d_{\text{H7-H8}}$	2.447
	$d_{\text{H7-H9}}$	2.459			$d_{\text{H10-H12}}$	2.446
	$d_{\text{H11-H13}}$	2.453			$d_{\text{H11-H13}}$	2.466
	$d_{\text{H12-H14}}$	2.448			$d_{\text{H13-H14}}$	2.548
	$d_{\text{H15-H17}}$	2.429			$d_{\text{H14-H15}}$	2.443
	$d_{\text{H16-H18}}$	2.454			$d_{\text{C1-C4}}$	2.443
	$d_{\text{C1-C4}}$	2.444			$d_{\text{C1-C5}}$	2.484
	$d_{\text{C1-C5}}$	2.481			$d_{\text{C2-C3}}$	2.398
	$d_{\text{C2-C3}}$	2.396			$d_{\text{C2-C6}}$	2.484
	$d_{\text{C2-C6}}$	2.481			$d_{\text{C3-C6}}$	2.444
	$d_{\text{C3-C6}}$	2.443			$d_{\text{C4-C5}}$	2.397
	$d_{\text{C4-C5}}$	2.396			$d_{\text{C7-C10}}$	2.458
	$d_{\text{C7-C10}}$	2.44			$d_{\text{C7-C12}}$	2.499
	$d_{\text{C7-C12}}$	2.476			$d_{\text{C8-C9}}$	2.401
	$d_{\text{C8-C9}}$	2.409			$d_{\text{C8-C11}}$	2.496
	$d_{\text{C8-C11}}$	2.496			$d_{\text{C9-C11}}$	2.465
	$d_{\text{C9-C11}}$	2.462			$d_{\text{C10-C12}}$	2.397
2.43	$d_{\text{C10-C12}}$	2.403		2.43	$d_{\text{C13-C16}}$	2.47
	$d_{\text{C13-C16}}$	2.467			$d_{\text{C13-C17}}$	2.506
	$d_{\text{C13-C17}}$	2.506			$d_{\text{C14-C18}}$	2.447
	$d_{\text{C14-C18}}$	2.508			$d_{\text{C14-C15}}$	2.501
	$d_{\text{C14-C15}}$	2.443			$d_{\text{C15-C18}}$	2.464
	$d_{\text{C15-C18}}$	2.468			$d_{\text{C16-C17}}$	2.451
	$d_{\text{C16-C17}}$	2.442			$d_{\text{C19-C24}}$	2.503
	$d_{\text{C19-C24}}$	2.498			$d_{\text{C19-C22}}$	2.469
	$d_{\text{C19-C22}}$	2.463			$d_{\text{C20-C21}}$	2.395
	$d_{\text{C20-C21}}$	2.401			$d_{\text{C20-C23}}$	2.473
	$d_{\text{C20-C23}}$	2.478			$d_{\text{C21-C23}}$	2.442
	$d_{\text{C21-C23}}$	2.44			$d_{\text{C22-C24}}$	2.403
	$d_{\text{C22-C24}}$	2.407			$d_{\text{C4-N1}}$	2.407
	$d_{\text{C4-N1}}$	2.421			$d_{\text{C9-N1}}$	2.44
	$d_{\text{C9-N1}}$	2.417			$d_{\text{C5-N1}}$	2.535
	$d_{\text{C5-N1}}$	2.522			$d_{\text{C10-N2}}$	2.384
	$d_{\text{C10-N2}}$	2.383			$d_{\text{C15-N2}}$	2.365
	$d_{\text{C15-N2}}$	2.364			$d_{\text{C16-N3}}$	2.366
	$d_{\text{C16-N3}}$	2.363			$d_{\text{C21-N3}}$	2.421
	$d_{\text{C21-N3}}$	2.377			$d_{\text{C22-N4}}$	2.416
	$d_{\text{C22-N4}}$	2.413			$d_{\text{O1-O2}}$	2.467
					$d_{\text{O1-O3}}$	2.479
					$d_{\text{O2-O3}}$	2.478
					$d_{\text{O4-O5}}$	2.483
					$d_{\text{O4-O6}}$	2.473
					$d_{\text{O5-O6}}$	2.471
					$d_{\text{Na1-O2}}$	2.462
					$d_{\text{Na2-O4}}$	2.461

	d_{C3-C4}	2.745		d_{C3-C4}	2.744
	d_{C9-C10}	2.753		d_{C9-C10}	2.749
	d_{C8-C12}	2.809		d_{C8-C12}	2.8
	$d_{C15-C16}$	2.77		$d_{C15-C16}$	2.778
	$d_{C14-C17}$	2.833		$d_{C14-C17}$	2.837
2.77	$d_{C21-C22}$	2.749	2.77	$d_{C21-C22}$	2.752
	$d_{C20-C24}$	2.808		$d_{C20-C24}$	2.798
	d_{B1-N1}	2.86		d_{B1-N1}	2.859
	d_{B2-N1}	2.854		d_{B2-N1}	2.859
	d_{B2-N2}	2.841		d_{B2-N2}	2.848
	d_{Q1-N2}	2.807		d_{Q1-N2}	2.807
	d_{Q1-N3}	2.808		d_{Q1-N3}	2.793
	d_{B3-N3}	2.843		d_{B3-N3}	2.836
	d_{B3-N4}	2.854		d_{B3-N4}	2.858
	d_{C1-H4}	3.417		d_{C1-H4}	3.417
	d_{C3-H2}	3.401		d_{C3-H2}	3.406
	d_{C6-H3}	3.416		d_{C6-H3}	3.417
	d_{C4-H5}	3.401		d_{C4-H5}	3.404
	d_{C5-H4}	3.392		d_{C5-H4}	3.39
	d_{C6-H2}	3.444		d_{C6-H2}	3.445
	d_{C2-H3}	3.392		d_{C2-H3}	3.394
	d_{C1-H5}	3.447		d_{C1-H5}	3.446
	d_{C8-H8}	3.402		d_{C7-H8}	3.436
	d_{C9-H7}	3.412		d_{C9-H7}	3.404
	d_{C12-H9}	3.387		d_{C12-H8}	3.384
	$d_{C10-H10}$	3.407		d_{C10-H9}	3.409
	d_{C7-H10}	3.444		d_{C7-H9}	3.449
	d_{C11-H8}	3.44		d_{C11-H7}	3.459
3.41	$d_{C15-H11}$	3.446	3.41	$d_{C15-H10}$	3.451
	$d_{C18-H11}$	3.48		$d_{C18-H10}$	3.469
	$d_{C14-H12}$	3.42		$d_{C14-H11}$	3.425
	$d_{C18-H12}$	3.458		$d_{C18-H11}$	3.456
	$d_{C17-H13}$	3.419		$d_{C17-H12}$	3.428
	$d_{C13-H13}$	3.458		$d_{C13-H12}$	3.262
	$d_{C16-H14}$	3.446		$d_{C16-H13}$	3.45
	$d_{C13-H14}$	3.476		$d_{C13-H13}$	3.484
	$d_{C23-H15}$	3.443		$d_{C23-H14}$	3.447
	$d_{C21-H15}$	3.408		$d_{C21-H14}$	3.396
	$d_{C20-H16}$	3.385		$d_{C22-H16}$	3.414
	$d_{C23-H16}$	3.423		$d_{C19-H16}$	3.452
	$d_{C24-H17}$	3.401		$d_{C19-H15}$	3.443
	$d_{C19-H17}$	3.44		$d_{C24-H15}$	3.4
	$d_{C19-H18}$	3.463			
	$d_{C24-H17}$	3.412			

	$d_{C2\text{-}N1}$	3.733		$d_{C2\text{-}N1}$	3.723
	$d_{C3\text{-}N1}$	3.777		$d_{C3\text{-}N1}$	3.784
	$d_{C12\text{-}N1}$	3.72		$d_{C12\text{-}N1}$	3.739
	$d_{C10\text{-}N1}$	3.768		$d_{C10\text{-}N1}$	3.764
	$d_{C9\text{-}N2}$	3.78		$d_{C9\text{-}N2}$	3.785
	$d_{C8\text{-}N2}$	3.698		$d_{C8\text{-}N2}$	3.697
3.71	$d_{C16\text{-}N2}$	3.742	3.71	$d_{C16\text{-}N2}$	3.747
	$d_{C17\text{-}N2}$	3.655		$d_{C17\text{-}N2}$	3.656
	$d_{C14\text{-}N3}$	3.655		$d_{C14\text{-}N3}$	3.65
	$d_{C15\text{-}N3}$	3.745		$d_{C15\text{-}N3}$	3.723
	$d_{C24\text{-}N3}$	3.695		$d_{C24\text{-}N3}$	3.721
	$d_{C22\text{-}N3}$	3.785		$d_{C22\text{-}N3}$	3.748
	$d_{C20\text{-}N4}$	3.717		$d_{C20\text{-}N4}$	3.719
	$d_{C21\text{-}N4}$	3.77		$d_{C21\text{-}N4}$	3.774
	$d_{C5\text{-}H2}$	3.891		$d_{C5\text{-}H2}$	3.894
	$d_{C3\text{-}H4}$	3.838		$d_{C3\text{-}H4}$	3.735
	$d_{C2\text{-}H5}$	3.892		$d_{C2\text{-}H5}$	3.894
	$d_{C4\text{-}H3}$	3.833		$d_{C4\text{-}H3}$	3.837
	$d_{C10\text{-}H8}$	3.846		$d_{C9\text{-}H8}$	3.841
	$d_{C12\text{-}H7}$	3.897		$d_{C12\text{-}H7}$	3.887
3.89	$d_{C9\text{-}H9}$	3.843	3.87	$d_{C8\text{-}H9}$	3.884
	$d_{C8\text{-}H10}$	3.897		$d_{C14\text{-}H13}$	3.928
	$d_{C14\text{-}H14}$	3.922		$d_{C15\text{-}H12}$	3.869
	$d_{C15\text{-}H13}$	3.861		$d_{C17\text{-}H10}$	3.925
	$d_{C17\text{-}H11}$	3.923		$d_{C16\text{-}H11}$	3.87
	$d_{C16\text{-}H12}$	3.861		$d_{C20\text{-}H16}$	3.882
	$d_{C20\text{-}H18}$	3.896		$d_{C24\text{-}H14}$	3.889
	$d_{C24\text{-}H15}$	3.896		$d_{C21\text{-}H15}$	3.846
	$d_{C21\text{-}H17}$	3.843			
	$d_{C22\text{-}H16}$	3.84			
	$d_{H4\text{-}H8}$	4.369		$d_{H3\text{-}H7}$	4.386
	$d_{H3\text{-}H7}$	4.394		$d_{H8\text{-}H11}$	4.172
	$d_{H12\text{-}H15}$	4.393		$d_{H11\text{-}H14}$	4.668
	$d_{H8\text{-}H11}$	4.375		$d_{C13\text{-}N3}$	4.269
	$d_{H12\text{-}H15}$	4.393		$d_{C11\text{-}N1}$	4.331
4.33	$d_{C13\text{-}N3}$	4.287	4.33	$d_{C1\text{-}N1}$	4.31
	$d_{C11\text{-}N1}$	4.319		$d_{C7\text{-}N2}$	4.317
	$d_{C1\text{-}N1}$	4.311		$d_{C18\text{-}N2}$	4.279
	$d_{C7\text{-}N2}$	4.29		$d_{C23\text{-}N3}$	4.284
	$d_{C18\text{-}N2}$	4.286		$d_{C19\text{-}N4}$	4.331
	$d_{C23\text{-}N3}$	4.293			
	$d_{C19\text{-}N4}$	4.321			

	$d_{C3\text{-}C7}$	4.681		$d_{C3\text{-}C7}$	4.693
	$d_{C4\text{-}C8}$	4.639		$d_{C4\text{-}C8}$	4.639
	$d_{C5\text{-}C9}$	4.641		$d_{C5\text{-}C9}$	4.693
	$d_{C6\text{-}C10}$	4.673		$d_{C6\text{-}C10}$	4.669
	$d_{C9\text{-}C13}$	4.589		$d_{C9\text{-}C13}$	4.59
	$d_{C10\text{-}C14}$	4.566		$d_{C10\text{-}C14}$	4.596
	$d_{C12\text{-}C15}$	4.606		$d_{C12\text{-}C15}$	4.615
4.59	$d_{N3\text{-}H12}$	4.663	4.59	$d_{N3\text{-}H11}$	4.638
	$d_{N1\text{-}H3}$	4.656		$d_{N1\text{-}H3}$	4.667
	$d_{H2\text{-}H6}$	4.506		$d_{H2\text{-}H6}$	4.462
	$d_{N1\text{-}H9}$	4.662		$d_{N1\text{-}H9}$	4.558
	$d_{C11\text{-}C16}$	4.571		$d_{C11\text{-}C16}$	4.573
	$d_{C9\text{-}C13}$	4.589		$d_{C9\text{-}C13}$	4.59
	$d_{C17\text{-}C21}$	4.592		$d_{C17\text{-}C21}$	4.471
	$d_{C18\text{-}C22}$	4.601		$d_{C18\text{-}C22}$	4.587
	$d_{C15\text{-}C19}$	4.583		$d_{C15\text{-}C19}$	4.549
	$d_{C10\text{-}C14}$	4.566		$d_{C10\text{-}C14}$	4.596
	$d_{H2\text{-}H5}$	4.979		$d_{H2\text{-}H5}$	4.981
	$d_{H3\text{-}H4}$	4.931		$d_{H3\text{-}H4}$	4.929
	$d_{H8\text{-}H9}$	4.937		$d_{H7\text{-}H9}$	4.971
	$d_{H7\text{-}H10}$	4.986		$d_{H10\text{-}H13}$	5.016
4.97	$d_{H11\text{-}H14}$	5.013	4.97	$d_{H11\text{-}H12}$	4.961
	$d_{H12\text{-}H13}$	4.952		$d_{H14\text{-}H16}$	4.973
	$d_{H15\text{-}H18}$	4.985			
	$d_{H16\text{-}H17}$	4.934			
